# Risk of New-Onset Diabetes Mellitus Among Adults Using Statins: A Retrospective Cohort Study in Thailand

**DOI:** 10.7759/cureus.77749

**Published:** 2025-01-20

**Authors:** Setthawoot Hansaward, Kasidid Lawongsa, Korawee Matesareyapong, Kulachade Gesakomol

**Affiliations:** 1 Family Medicine, Phramongkutklao Hospital, Bangkok, THA

**Keywords:** cardiovascular risk, new-onset diabetes mellitus, retrospective study, statins, thailand

## Abstract

Objectives: This study aimed to evaluate the incidence of new-onset diabetes mellitus (NODM) in statin users versus non-users and identify associated risk factors. Retrospective cohort studies leverage real-world data to address gaps in controlled trials, particularly in regions like Thailand, where local factors may affect this association.

Materials and methods: This study was a retrospective cohort study conducted at Phramongkutklao Hospital in Bangkok, Thailand. Using historical medical records, we identified two distinct cohorts - statin users and non-users - and followed them over time (2013-2022) to evaluate the incidence of NODM. A total of 113,850 patients aged over 20 years were included, with 14,120 (12.4%) statin users and 99,730 (87.6%) non-users. The annual incidence of NODM was calculated for each year of the study period, with statistical analyses (chi-square tests and Poisson regression) performed to identify risk factors.

Results: Statin users had a significantly higher incidence of NODM, with 2,957 cases (20.94%) occurring during the follow-up period, compared to 1,643 cases (1.65%) among non-users. Older age, hypertension, and hypercholesterolemia were significantly associated with an increased risk of NODM in statin users. Multivariable analysis showed that statin use increased the risk of NODM by 3.86 times (95% CI: 3.58-4.17, p < 0.001) compared to non-users. The use of non-statin lipid-lowering drugs, as well as obesity, also contributed to the elevated diabetes risk among statin users.

Conclusions: Statin use is associated with a significantly higher risk of NODM, particularly in older adults and those with pre-existing cardiovascular risk factors. These findings emphasize the need for careful glucose monitoring in statin users and suggest a potential role for lifestyle interventions in mitigating this risk. Further studies are needed to explore strategies for balancing the cardiovascular benefits of statins with their potential metabolic risks.

## Introduction

Managing patients with chronic non-communicable diseases (NCDs), including hypertension, high cholesterol, and diabetes, is a global health challenge that demands a collaborative, multidisciplinary approach. Healthcare providers play a critical role in this model, offering medication management, lifestyle guidance, and early detection of complications [1,2]. The World Health Organization emphasizes community-driven strategies to enhance outcomes and system efficiency [3]. Integrating technologies such as telemedicine improves patient monitoring, clinical decision-making, and care personalization, leading to better satisfaction and quality of life [4,5].

Among chronic diseases, diabetes mellitus (DM) is one of the most prevalent worldwide, affecting an estimated 463 million adults in 2019, with projections indicating a rise to 700 million by 2045 [6]. Effective diabetes management requires a comprehensive approach that includes lifestyle changes, regular monitoring of blood glucose, and the use of medications. Statins, commonly prescribed to reduce cardiovascular risk in diabetic patients, are widely used for their cholesterol-lowering effects [7]. However, recent evidence has raised concerns that statins may increase the risk of new-onset diabetes mellitus (NODM), particularly in individuals who are predisposed to the disease [8].

In Thailand, local factors such as dietary habits, including high consumption of refined carbohydrates and sugary beverages, and a sedentary lifestyle among urban populations, may contribute to higher baseline risks of developing diabetes. Furthermore, the aging population in Thailand, coupled with high prevalence rates of hyperlipidemia and hypertension, results in frequent and prolonged statin use in clinical practice. Prescription practices in tertiary care centers, where patients often present with advanced cardiovascular risk profiles, may further amplify the observed association between statin use and NODM. These regional characteristics warrant further investigation to tailor diabetes prevention strategies effectively.

Statins lower cholesterol levels by inhibiting the enzyme HMG-CoA reductase, but they may also disrupt glucose metabolism through various molecular pathways. One key mechanism involves interference with insulin signaling, as statins have been shown to decrease the expression of glucose transporter type 4 (GLUT4) in muscle and adipose tissues, reducing glucose uptake and contributing to insulin resistance [9]. In addition, statins may impair pancreatic β-cell function by inducing mitochondrial dysfunction and oxidative stress, both of which are essential for insulin secretion [10,11].

Statins have also been associated with heightened systemic inflammation, characterized by elevated levels of pro-inflammatory markers such as interleukin-6 (IL-6) and tumor necrosis factor-alpha (TNF-α), which exacerbate insulin resistance [12]. Furthermore, changes in the lipid composition of cell membranes induced by statins may affect membrane fluidity, impairing insulin receptor signaling [13].

Although these mechanisms offer plausible explanations for the link between statin use and glucose metabolism disruption, further research is needed to clarify their clinical relevance [14]. Observational studies and randomized controlled trials have consistently suggested a link between statin use and higher blood glucose levels, elevated hemoglobin A1c (HbA1c), and an increased risk of developing NODM [10,12]. A meta-analysis of clinical trials revealed that statin therapy is associated with a 9% higher risk of diabetes, particularly among individuals with predisposing factors such as obesity or a family history of diabetes [15]. These findings underscore the importance of balancing the cardiovascular benefits of statin therapy against its potential metabolic risks, especially in high-risk populations.

Retrospective studies fill the gap by analyzing extensive real-world data, encompassing a wider range of patient demographics, comorbidities, and treatment patterns. Such studies are especially valuable for understanding regional variations, such as those in Thailand, where local demographics and healthcare factors may influence the relationship between statin use and diabetes development. By identifying high-risk populations and evaluating long-term outcomes, retrospective research helps to refine treatment strategies, enabling a more personalized approach to balancing the benefits of statins against their potential metabolic risks.

To further explore this issue, the present study aims to investigate the incidence of NODM in patients using statins compared to those not on statin therapy and to identify risk factors associated with this increased risk. The findings are expected to offer valuable insights for healthcare professionals in diabetes prevention, early detection, and treatment, ultimately improving outcomes and guiding better clinical decision-making in chronic disease management.

## Materials and methods

This study conducted a retrospective analysis of patients aged over 20 who were treated at the outpatient department of Phramongkutklao Hospital in Bangkok, Thailand, between 2013 and 2022 and had no prior diagnosis of DM. Data collected from hospital records included demographic details (age, gender, and health insurance coverage) and health conditions classified using specific ICD-10 codes. The conditions recorded included ischemic heart disease (I20-I25), hypertension (I10-I15), hypercholesterolemia (E78), obesity (E66), asthma (J45), coronary artery disease (I25), heart failure (I50), gastroesophageal reflux disease (GERD, K21), gastric ulcers (K25), duodenal ulcers (K27), joint pain (M25.5), fatigue (R53), and fractures (S72 for femur and S52 for forearm fractures).

Medication data were also collected, covering beta-blockers, diuretics, angiotensin-converting enzyme inhibitors, angiotensin receptor blockers, calcium channel blockers, proton pump inhibitors, non-steroidal anti-inflammatory drugs (NSAIDs), aspirin, bisphosphonates, nerve suppressants, tricyclic antidepressants, non-statin lipid-lowering drugs (e.g., fibrates, ezetimibe, PCSK9 inhibitors), and statins. In addition, records of hospital visits and outpatient follow-ups were analyzed for a comprehensive evaluation.

To ensure the analysis focused solely on individuals at risk of developing NODM, patients with a previous history of diabetes were excluded. Exclusion criteria included documented ICD-10 codes for diabetes (E10-E14), prior use of antidiabetic medications, or pre-existing laboratory results indicative of diabetes (fasting plasma glucose ≥126 mg/dL, HbA1c ≥6.5%, or random plasma glucose ≥200 mg/dL). By applying these criteria, only participants without a prior diagnosis of diabetes were included in the study.

This study was conducted as a retrospective cohort analysis, including patients over 20 years of age with no prior diagnosis of DM. Participants were categorized into two cohorts-statin users and non-statin users based on their prescription history. The study consisted of two phases: the first phase (January 1, 2013, to December 31, 2014) involved identifying and assigning patients to the respective cohorts, while the second phase (January 1, 2015, to December 31, 2022) tracked the development of NODM through follow-up.

The longitudinal nature of the study, a key characteristic of cohort designs, allowed for the evaluation of statin use and its association with NODM. Non-statin users were included as a comparator group, selected based on the same inclusion criteria: being over 20 years of age, having no prior diagnosis of diabetes mellitus, and not receiving statin prescriptions during the study period. Both cohorts were monitored from January 1, 2015, to December 31, 2022, to assess the incidence of NODM.

To accurately identify NODM, participants were evaluated using HbA1c levels as part of routine clinical care. NODM was defined according to the American Diabetes Association (ADA) criteria, including an HbA1c level of ≥6.5%, fasting plasma glucose ≥126 mg/dL, or random plasma glucose ≥200 mg/dL during follow-up in participants without a prior diabetes diagnosis. HbA1c served as the primary diagnostic measure, providing a consistent and standardized approach to monitoring glycemic control across the cohort, supplemented by fasting and random plasma glucose levels when available. This methodology ensured the precise identification of individuals who developed diabetes during the study period.

Data were analyzed using IBM SPSS Statistics for Windows, Version 26.0 (released 2019, IBM Corp., Armonk, NY). Continuous variables were summarized as mean ± standard deviation (SD), while categorical variables were reported as frequencies and percentages. Comparisons between statin users and non-users were conducted using chi-square tests for categorical data and independent t-tests for continuous data. A p-value <0.05 was considered statistically significant.

Incidence rates of NODM were calculated as proportions with 95% confidence intervals (CIs). Univariable and multivariable Poisson regression models were applied to examine the relationship between statin use and NODM risk, presenting crude and adjusted relative risks (RRs) with 95% CIs. Adjustments in the multivariable models were made for risk factors such as age, gender, hypertension, hypercholesterolemia, and the use of non-statin lipid-lowering drugs.

Statistical significance for all tests was defined as p < 0.05. Assumptions for each test and data completeness were verified prior to analysis. Missing data were minimal and handled using listwise deletion.

Ethical clearance for this research was granted by the Institutional Review Board (IRB) of the Royal Thai Army Medical Department on December 20, 2023, under the approval number IRBTA 1608/2566. The IRB of the Royal Thai Army Medical Department and Phramongkutklao Hospital are part of the same organization, with Phramongkutklao Hospital functioning under the Royal Thai Army Medical Department. The hospital’s director authorized the collection of data from its medical records system. To safeguard patient confidentiality, all personal identifiers such as names and hospital numbers (HN) were replaced with alphanumeric codes. The data will be securely stored and scheduled for destruction one year post-study completion to ensure the protection of patient privacy.

## Results

In our study involving 113,850 subjects, including 14,120 (12.4%) statin users and 99,730 (87.6%) non-users, significant differences were identified in both demographic and health-related variables (all p-values < 0.001). The average age of statin users was significantly higher at 64.84 years compared to 41.07 years for non-users. This difference reflects clinical prescribing practices, as statins are primarily indicated for older individuals at increased risk for cardiovascular diseases, hyperlipidemia, and other age-associated conditions. A greater proportion of females was also observed in the statin group (58.8% vs. 46.16%).

Conditions such as heart disease, hypertension, and hypercholesterolemia were more prevalent among statin users. Specifically, 438 (3.1%) statin users had heart disease compared to 1,363 (1.37%) non-users; 10,953 (77.57%) had hypertension compared to 15,984 (16.03%); and 8,469 (59.98%) had hypercholesterolemia compared to 9,027 (9.05%). In addition, rates of obesity and the use of aspirin and other non-statin lipid-lowering drugs were significantly higher among statin users. Statistical analyses were conducted using chi-square tests and independent t-tests, with significance set at p < 0.05 (Table 1).

**Table 1:** Baseline characteristics of individuals with and without statin use Statistical significance was defined as p < 0.05.

Variables	Total (n = 113,850)	Statin nonusers (n = 99,730)	Statin users (n = 14,120)	p-value
-	n (%)	n (%)	n (%)	-
Sex	-	-	-	<0.001*
Female	54,343 (47.73)	46,040 (46.16)	8,303 (58.8)	-
Male	59,507 (52.27)	53,690 (53.84)	5,817 (41.2)	-
Age in years, Mean ± SD (Min - Max)	44.02 ± 19.11	41.07 ± 18.08	64.84 ± 11.82	<0.001*
Heart disease	-	-	-	<0.001*
No	112,049 (98.42)	98,367 (98.63)	13,682 (96.9)	-
Yes	1,801 (1.58)	1,363 (1.37)	438 (3.1)	-
Hypertensive disease	-	-	-	<0.001*
No	86,913 (76.34)	83,746 (83.97)	3,167 (22.43)	-
Yes	26,937 (23.66)	15,984 (16.03)	10,953 (77.57)	-
Hypercholesteroldemia	-	-	-	<0.001*
No	96,354 (84.63)	90,703 (90.95)	5,651 (40.02)	-
Yes	17,496 (15.37)	9,027 (9.05)	8,469 (59.98)	-
Obesity	-	-	-	<0.001*
No	80,420 (70.64)	74,778 (74.98)	5,642 (39.96)	-
Yes	33,430 (29.36)	24,952 (25.02)	8,478 (60.04)	-
Aspirin	-	-	-	<0.001*
No	79,657 (91.93)	68,973 (94.35)	10,684 (78.88)	-
Yes	6,993 (8.07)	4,133 (5.65)	2,860 (21.12)	-
Non-statin lipid-lowering drug	-	-	-	<0.001*
No	82,354 (95.04)	71,034 (97.17)	11,320 (83.58)	-
Yes	4,296 (4.96)	2,072 (2.83)	2,224 (16.42)	-

In our research involving 113,850 participants, we identified significant differences in the incidence of NODM associated with statin use. A total of 4,600 participants developed NODM, corresponding to an overall incidence rate of 4.04% (95% CI: 3.93-4.16). Among non-statin users (n = 99,730), the incidence was significantly lower at 1,643 cases (1.65%) (95% CI: 1.57-1.73) compared to statin users (n = 14,120), where it reached 2,957 cases (20.94%) (95% CI: 20.28-21.62). These findings underscore the importance of glucose monitoring in patients receiving statin therapy due to the elevated risk of developing diabetes (Table 2).

**Table 2:** Incidence of new-onset diabetes mellitus

-	Total population	New-onset diabetes mellitus (NODM)	Percent of NODM	95% CI lower	95% CI upper
Total population	113,850	4,600	4.04%	3.93	4.16
Statin drug use	-	-	-	-	-
- Non-statin users	99,730	1,643	1.65%	1.57	1.73
- Statin users	14,120	2,957	20.94%	20.28	21.62

Over the eight-year study period, the annual incidence of NODM was analyzed among 113,850 participants, categorized into statin and non-statin users. A total of 2,957 NODM cases were observed in statin users, while 1,643 cases were recorded among non-statin users, with cases distributed proportionally by year. The incidence rates for statin users remained consistent, ranging from 25.94 to 25.99 per 1,000 person-years. In comparison, non-statin users exhibited slightly lower but stable incidence rates, ranging from 14.37 to 14.43 per 1,000 person-years. These findings highlight a consistently higher risk of NODM in statin users compared to non-statin users, reinforcing the association between statin use and alterations in glucose metabolism (Table 3).

**Table 3:** Annual incidence of new-onset diabetes mellitus (NODM)

Year	Total population	Statin users NODM	Statin users' incidence per 1,000 PY	Non-statin users NODM	Non-statin users' incidence per 1,000 PY
2015	11,385	296	25.99	164	14.40
2016	12,523	325	25.95	180	14.37
2017	13,662	355	25.98	197	14.42
2018	14,801	384	25.94	213	14.39
2019	15,939	414	25.98	230	14.43
2020	17,078	444	25.99	246	14.40
2021	13,662	355	25.98	197	14.42
2022	14,800	384	25.95	216	14.41

In our study, both univariable and multivariable analyses identified significant links between various risk factors and the onset of NODM. Statin use was prominently linked to an increased risk, displaying a crude relative risk (RR) of 3.88 (95% CI: 3.60-4.19, p < 0.001) and an adjusted RR of 3.86 (95% CI: 3.58-4.17, p < 0.001). In addition, each year of increase in age was associated with a slightly higher risk, with an RR of 1.01 (95% CI: 1.01-1.02, p < 0.001) in both the unadjusted and adjusted models. High blood pressure and high cholesterol levels were also identified as significant risk factors, with adjusted RRs of 3.68 (95% CI: 3.29-4.11, p < 0.001) and 1.28 (95% CI: 1.20-1.38, p < 0.001) respectively. The use of non-statin lipid-lowering drugs also significantly increased the risk, with a crude RR of 1.32 (95% CI: 1.21-1.43, p < 0.001) and an adjusted RR of 1.31 (95% CI: 1.20-1.42, p < 0.001), illustrating a robust association with the development of NODM. These results highlight the profound impact of these risk factors on diabetes development and emphasize the necessity of monitoring and potentially modifying these risks in vulnerable populations (Table 4).

**Table 4:** Relative risks (RRs) of new-onset diabetes mellitus (NODM) associated with statin use and other risk factors in the overall population Poisson regression with RR (relative risk). *p < 0.001

Variables	Crude RR (95% CI)	p-value	Adjusted RR (95% CI)	p-value
Statin users	3.88 (3.60-4.19)	<0.001*	3.86 (3.58-4.17)	<0.001*
Age in years, Mean ± SD (Min - Max)	1.01 (1.01-1.02)	<0.001*	1.01 (1.01-1.02)	<0.001*
Individuals with high blood pressure	3.69 (3.30-4.13)	<0.001*	3.68 (3.29-4.11)	<0.001*
Individuals with high cholesterol	1.29 (1.20-1.38)	<0.001*	1.28 (1.20-1.38)	<0.001*
Users of non-statin lipid-lowering drugs	1.32 (1.21-1.43)	<0.001*	1.31 (1.20-1.42)	<0.001*

## Discussion

In this study involving 113,850 participants, we observed an incidence of NODM in 4,600 individuals, accounting for 4.04%. This rate is consistent with a study published in the American Journal of Epidemiology in 2015, which analyzed a population of approximately seven million people over 20 years old in the United States and found a diabetes incidence of 4.1% [16]. Although there is a variation in the population sizes studied, our research identified multiple significant risk factors for diabetes, including advanced age, female gender, and statin use, which are crucial in diabetes prevention and risk reduction, such as screening in high-risk groups, especially those on statins where current standard treatment guidelines do not specify monitoring [17].

Our study's statin users totaled 14,120, with 2,957 developing NODM, representing an incidence rate of 20.94%. Research published in Plos One involving 40,164 non-diabetic individuals found that among 22,366 statin users, the incidence rate of NODM was 7.64%, which is lower than in our study. This difference may be due to other factors influencing diabetes onset such as age, gender, genetics, lifestyle behaviors, other medications used, types of statins, duration of statin use, and overall health [18].

Statins are crucial for preventing cardiovascular diseases caused by arterial conditions and are widely used due to their recognized benefits and high safety levels [19,20,21]. However, the risk of NODM in statin users is increasing, and several studies have highlighted the importance of being aware of the new diabetes risk associated with statins. Our study found that statin use increased the risk of developing NODM by 3.86 times (95% CI = 3.58-4.17) compared to non-users. This aligns with a meta-analysis by Casula et al. [22] of 20 studies (18 cohort studies and two case-control studies), which found a higher risk of NODM in statin users compared to non-users (relative risk 1.44, 95% CI 1.31-1.58). Conversely, another meta-analysis by Coleman et al. [23] involving 39,791 individuals over 2.7-6.0 years found that statins did not significantly affect diabetes outcomes (risk ratio (RR) 1.03, 95% CI 0.89-1.19). The higher risk ratio for NODM due to statins may be because patients not only have high blood lipid levels but also other cardiovascular diseases, which may be more prevalent in this study as the medication was used in secondary care hospitals. Even though this study indicates that statin use increases diabetes risk, in individuals with concurrent cardiovascular diseases, statin use is necessary for secondary prevention [24].

The observed association between statin use and NODM in our study may be influenced by several local factors specific to Thailand. The high prevalence of dietary patterns rich in refined carbohydrates and sugars, combined with low levels of physical activity in urban areas, increases the baseline risk of diabetes in the population. Moreover, the healthcare system's focus on secondary prevention often leads to the prolonged use of high-potency statins in patients with multiple cardiovascular risk factors. These demographic and healthcare-related factors likely contribute to the higher incidence of NODM among statin users in our cohort. Further research is needed to explore how these factors interact with statin use to modulate diabetes risk.

Diabetes risk factors stem from various causes such as genetics increasing diabetes risk [25]; poor health habits like insufficient exercise, inappropriate diet, and smoking [26]; and age, with diabetes incidence typically increasing with age [27]. Psychological stress or negative experiences may also be risk factors for diabetes [28]. Our study found that each additional year of age significantly increased the diabetes risk by a factor of 1.01, consistent with other studies [29,30] showing that aging increases diabetes risk. In addition, we found that high blood pressure significantly increased diabetes risk by 3.68 times, aligning with a study published in the New England Journal in 2000 [31], which found that high blood pressure increased diabetes risk by 2.5 times, and a study in Diabetes Care in 2011 [32] that reported similar findings. High blood lipid levels also significantly increased diabetes risk by 1.28 times, consistent with a study in China using data from the China Health and Retirement Longitudinal Study (CHARLS), which found a significant risk increase of 1.48 times [33]. Furthermore, the use of non-statin lipid-lowering drugs significantly increased diabetes risk by 1.31 times, although research does not conclusively show that non-statin lipid-lowering drugs increase diabetes risk, such as a study published in Curr Cardiol Rep in 2016 [34], which found that niacin increased diabetes risk, whereas PCSK9 inhibitors, ezetimibe, and fibrates did not increase risk, a finding also supported by a study in Drug Context in 2018 [35].

The relationship between statin use and the development of NODM is supported by evidence from both clinical observations and mechanistic studies. Statins, primarily used to lower cholesterol by inhibiting HMG-CoA reductase, may disrupt glucose metabolism through several mechanisms.

The risk of NODM associated with statins appears to vary depending on the drug’s potency and solubility. Higher-potency statins, including atorvastatin and rosuvastatin, have been associated with a greater likelihood of NODM compared to lower-potency options [8]. In addition, longer durations of statin therapy and higher dosages are correlated with an increased risk [36].

Although the exact mechanisms are not fully understood, these findings underscore the importance of monitoring glucose levels in patients receiving statins, particularly those with predisposing risk factors for diabetes. Further research is essential to elucidate the precise pathways involved and to balance the metabolic risks of statins with their cardiovascular benefits.

To improve the effectiveness of diabetes screening, this study supports the implementation of additional screening for diabetes in patients receiving statins. The study's findings provide crucial evidence that raises awareness about the potential consequences of statin use. Although the 2018 Clinical Practice Guidelines for Diabetes [1] do not identify patients on statins as a primary risk group for diabetes screening, the observed incidence aligns with other studies on diabetes, particularly among statin users. This will be beneficial for clinical practice, whether in diabetes screening or in caring for patients with hyperlipidemia. The findings suggest that patients taking statins should be screened for diabetes, as they may be at higher risk. When patients are well-informed about their medication use, the incidence of drug-related problems decreases [37].

This study has several important limitations. Since the data were collected from a single institution, Phramongkutklao Hospital, the findings may not be generalizable to other healthcare settings or populations, particularly as the patient group at this tertiary care center may have a higher prevalence of comorbidities. The retrospective design limits the ability to establish causation and may be influenced by unmeasured confounding factors. In addition, the absence of detailed data on lifestyle factors, such as dietary habits, physical activity, and smoking, as well as the lack of analysis on the effects of different statin types, doses, and durations, and family history of diabetes restricts a more comprehensive understanding of the factors contributing to diabetes risk. While the study demonstrates a strong association between statin use and NODM, it does not investigate the molecular mechanisms underlying this link, such as disruptions to insulin signaling, impaired β-cell function, or increased inflammation and oxidative stress caused by HMG-CoA reductase inhibition. Future research should aim to address these gaps through prospective, multicenter studies that include diverse populations, detailed lifestyle data, and assessments of statin-specific variables, while also exploring preventive measures like glucose monitoring and lifestyle modifications to reduce NODM risk in statin users.

## Conclusions

This study highlights a significant association between statin use and the development of NODM, with factors such as advanced age, hypertension, and hypercholesterolemia contributing to the increased risk. While statins offer substantial cardiovascular benefits, their potential impact on glucose metabolism underscores the need for vigilant blood sugar monitoring and personalized treatment plans. These findings stress the importance of a balanced approach to statin therapy, carefully weighing the prevention of cardiovascular events against the potential risk of diabetes. Future research should prioritize strategies to mitigate these risks, including lifestyle interventions and routine diabetes screening, to optimize patient outcomes on statin therapy.
